# Raman imaging of changes in the polysaccharides distribution in the cell wall during apple fruit development and senescence

**DOI:** 10.1007/s00425-015-2456-4

**Published:** 2016-01-05

**Authors:** Monika Szymańska-Chargot, Monika Chylińska, Piotr M. Pieczywek, Petra Rösch, Michael Schmitt, Jürgen Popp, Artur Zdunek

**Affiliations:** Institute of Agrophysics, Polish Academy of Sciences, ul. Doswiadczalna 4, 20-290 Lublin 27, Poland; Institute of Physical Chemistry and Abbe Center of Photonics, Friedrich Schiller University Jena, 07743 Jena, Germany; Leibniz Institute of Photonic Technology (IPHT), 07745 Jena, Germany

**Keywords:** Raman imaging, Cell wall, Apple ripening, Polysaccharides

## Abstract

**During on-tree ripening, the pectin distribution changed from polydispersed in cell wall to cumulated in cell wall corners. During apple storage, the pectin distribution returned to evenly dispersed along the cell wall.**

The plant cell wall influences the texture properties of fruit tissue for example apples become softer during ripening and postharvest storage. This softening process is believed to be mainly connected with changes in the cell wall composition due to polysaccharides undergoing an enzymatic degradation. These changes in polysaccharides are currently mainly investigated via chemical analysis or monoclonal labeling. Here, we propose the application of Raman microscopy for evaluating the changes in the polysaccharide distribution in the cell wall of apples during both ripening and postharvest storage. The apples were harvested 1 month and 2 weeks before optimal harvest date as well as at the optimal harvest date. The apples harvested at optimal harvest date were stored for 3 months. The Raman maps, as well as the chemical analysis were obtained for each harvest date and after 1, 2 and 3 months of storage, respectively. The analysis of the Raman maps showed that the pectins in the middle lamella and primary cell wall undergo a degradation. The changes in cellulose and hemicellulose were less pronounced. These findings were confirmed by the chemical analysis results. During development changes of pectins from a polydispersed form in the cell walls to a cumulated form in cell wall corners could be observed. In contrast after 3 months of apple storage we could observe an substantial pectin decrease. The obtained results demonstrate that Raman chemical imaging might be a very useful tool for a first identification of compositional changes in plant tissue during their development. The great advantage Raman microspectroscopy offers is the simultaneous localization and identification of polysaccharides within the cell wall and plant tissue.

**Electronic supplementary material:**

The online version of this article (doi:10.1007/s00425-015-2456-4) contains supplementary material, which is available to authorized users.

## Introduction

Apples are one of the most popular fruits among consumers all over the world (Konopacka et al. 2010). The softening of apples during ripening and postharvest storage is still a challenging problem affecting the quality and in turn strongly influences consumer acceptability (Billy et al. 2008; Ng et al. 2013). Many textural characteristics of fruit are related to its tissue microstructure and mechanical properties. These properties are determined by cell size, cell-to-cell adhesion, packing and turgor, wall thickness, wall composition and the reaction of cells to shearing stress (Volz et al. 2003; Harker et al. 2010; Li et al. 2013).

The plant cell wall structure and composition plays an important part in determining the fruit’s tissue structure and therefore the mechanical properties of the whole fruit. The plant cell wall structure not only provides the physical strength and maintains the cell shape continuously resisting the internal turgor pressure, but also regulates the cell morphogenesis and provides a barrier during exposure on biotic/abiotic stress (Hamann 2012; Cosgrove and Jarvis 2012). Therefore, to maintain functionality during the developmental growth the structure and composition of the cell wall is continuously modified (Xia et al. 2014). The most common model of a primary plant cell wall introduces the cell wall as a composite of a cellulose-hemicellulose network embedded in a pectin matrix (Carpita and Gibeaut 1993). Usually, plant cell walls comprise of 15–40 % cellulose, 30–50 % pectic polysaccharides and 20–30 % xyloglucan and lesser amounts of other hemicelluloses as arabinoxylans or glucomannans, and structural proteins (on a dry weight basis) (Cosgrove and Jarvis 2012). In case of apples, fully developed cells are rather large (approx. 0.1–0.3 mm diameter), turgid and thin-walled, and are loosely packed (airspace approx. 20–25 % of fruit volume). During the development and storage of an apple several changes of polysaccharides in the cell wall are connected with structural changes of pectins, hemicelluloses and cellulose, i.e., depolymerization, dissolution, but also with their spatial arrangement (Winisdorffer et al. 2015). All these changes lead to the degradation of cell wall polymers which causes a decrease of its integrity and in consequence a softening of the fruits. In the case of apples, the most pronounced changes are connected with the dynamics of enzymatically induced pectins degradation.

Other important features influencing the tissue microstructure is the cell-to-cell adhesion which is governed by the middle lamella and its dissolution during fruits on-tree and postharvest ripening (Ng et al. 2013; Jarvis et al. 2003). The middle lamella is formed by low-esterified galacturonic acid residues of homogalacturonan pectins and is interconnected by calcium bridges. The enzymatic dissolution of calcium crosslinks influences the integrity of the middle lamella and leads to a separation of adjacent cells (Cybulska et al. 2015).

Several microscopic techniques exist that might be useful to investigate the structural and functional changes of plant cell walls during the development and senescence of fruits. Among them the most popular are transmission electron microscopy TEM, confocal laser scanning microscopy CLSM, fluorescence microscopy, and atomic force microscopy AFM (Ng et al. 2015; Zdunek et al. 2014). However, none of these methods can provide detailed information about the chemical distribution, quantity and structural arrangement of the original building substances of the walls with a micrometer spatial resolution. Moreover, most of them require the extraction of cell wall polysaccharides and therefore lead to a destruction of the fruit. Nevertheless, there are confocal microscopy techniques which can be combined with a chemical analysis or immunolocalisation. These methods have been developed for an examination of the cell wall structure and composition, and thus for the observation of dynamic processes occurring therein. However, the required staining makes this technique relatively expensive. These obstacles can be overcome by employing confocal Raman microspectroscopy which provides information about the chemical composition and distribution of the individual substances in a label-free and non-invasive manner. The great advantage of Raman imaging is that all the polysaccharides are probed at the same time. However, the data analysis can be very challenging (Gierlinger et al. 2012). Raman imaging techniques have been successfully applied to observe the differences between various wood tissues, or to monitor changes within the distribution of active substances in other plants (Agarwal 2006; Gierlinger et al. 2008; Qin et al. 2011; Richter et al. 2011; Schmidt et al. 2009, 2010; Strehle et al. 2005). In our previous work, chemical Raman maps highlighting the pectin and cellulose polysaccharides distribution could be recorded for minimally prepared tomato samples (Chylińska et al. 2014).

So far the monitoring of distribution changes of the polysaccharides composition within the parenchyma cell walls of fruits and vegetables with respect to their development and ripening using Raman microspectroscopic have not been reported. Therefore, in this paper, the changes in the cell wall polysaccharides content, their distribution and structure during the on-tree maturation and storage of apple fruits were studied by means of Raman microspectroscopy. The Raman data were complemented by chemical analysis methods of the cell wall composition to obtain the most complete picture of the cell wall changes taking place during pre- and postharvest fruit ripening.

## Materials and methods

### Sample material

**Scheme 1:**
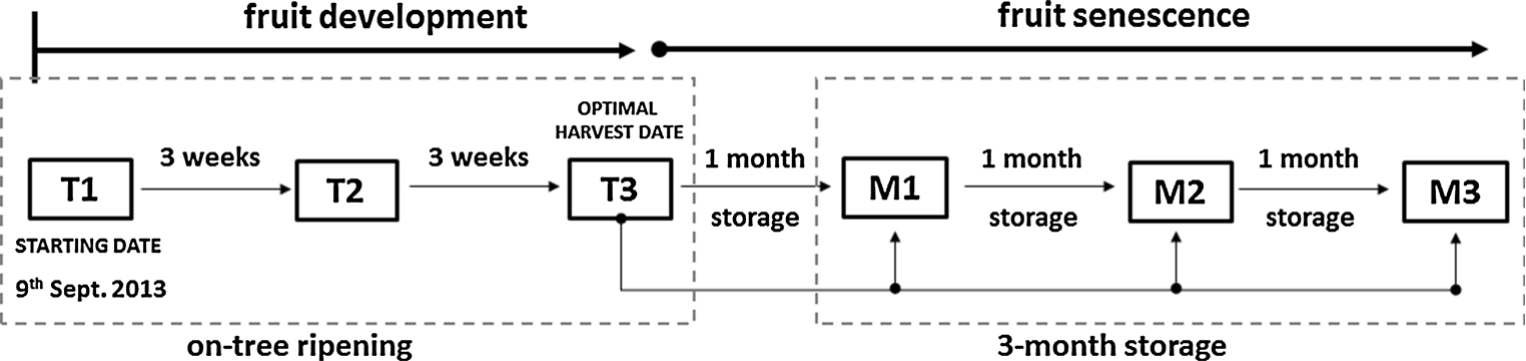
The scheme showing the sampling procedure

One cultivar of golden delicious apples (*Malus × domestica* cv. Golden Delicious) was chosen. Samples were taken at three different development stages T1–T3: before optimal harvest (T1 and T2) and at the optimal harvest date (T3). The time gap between the stages T1–T3 was 6 weeks, starting from beginning of September 2013. Only, apples collected at stage T3 were then stored in a cold room (4°) for 3 months. The sampling in details is presented on Scheme 1. It was ensured that the fruits were of similar size and blushing. For the experiments on stored apples the samples (cuts) were taken every month after harvest (M1, M2 and M3).

The fruits were cored and cut into smaller samples that were sliced by a vibratome (LEICA VT 1000S) in equatorial direction into slices of a thickness of 180 μm. The slices were placed without further preparation on a microscope slide covered with aluminum foil to avoid interference of the glass Raman bands with those of the plant cell wall. These samples placed on the microscope slides were left to dry on air.

## Analysis of the polysaccharide content

### Isolation of cell wall material

The cell wall material (CWM) was collected from the parenchyma tissue, without the skin and mesocarp to perform a chemical analysis of their composition. CWM was isolated using the modified hot alcohol-insoluble solids method as proposed by Renard (2005).

### Cellulose and hemicellulose content

The modified Van Soest’s method was used to determine the hemicellulose and cellulose content by a crude fiber extractor FIWE 3 (Velp Scientifica, Italy) (Szymańska-Chargot et al. 2015; Chylinska et al. 2016). Briefly, in this method, the cell wall samples were separated progressively into NDF (neutral detergent fiber) and ADF (acid detergent fiber) via extraction with neutral detergent solution (NDS) and acid detergent solution (ADS), respectively. The hemicellulose yield H was estimated as follows:

1 $${\text{H }} = \frac{{m_{\text{NDF}} - m_{\text{ADF}} }}{{m_{\text{CWM}} }} \cdot 100,$$

and the cellulose yield C results in:

2 $${\text{C }} = \frac{{m_{\text{ADF}} }}{{m_{\text{CWM}} }} \cdot 100,$$

where:

*m*_CWM_—weight of the cell wall material (CWM),

*m*_NDF_—weight of the neutral detergent fiber (NDF) left after extraction with NDS,

*m*_ADF_—weight of the acid detergent fiber (ADF) left after extraction with ADS.

For each sample the extraction was conducted in triplicate. All results are expressed as mg per g of cell wall material dry weight.

### Colorimetric quantification of galacturonic acid

To obtain information about the content of galacturonic acid (GalA) in pectic polysaccharide the isolated CWMs were further subjected to fractionation using the method proposed by Redgwell et al. (2008) with some modifications. Briefly, the CWM undergo sequential extraction by water, chelator and diluted alkali and in result the supernatants of water soluble pectins (WSP), chelator soluble pectins (CSP) and diluted alkali soluble pectins (DASP) are obtained (for details please see: Szymanska-Chargot and Zdunek 2013; Cybulska et al. 2015). The galacturonic acid content reflects the pectin content in the cell wall sample. Chemical analysis of galacturonic acid content in the WSP, CSP and DASP fractions was performed with an Automated Wet Chemistry Analyser (San++, Skalar Analytical, The Netherlands) (Szymanska-Chargot and Zdunek 2013; Cybulska et al. 2015). This is an automated procedure for colorimetric determination of galacturonic acid based on the total decomposition of a pectin sample in an acidic medium (hydrolysis with sulphuric acid). The obtained products are transformed to furfural derivatives, which, when reacting with 3-phenyl phenol, form a colored dye measured at 530 nm. The pectin content is expressed as galacturonic acid content in the dry weight of the cell wall material (mg/g). The sum of the obtained results provides a total galacturonic acid content for each sample. All the analyses were conducted in triplicate.

### Raman microscope and analysis of Raman images

The Raman spectra were taken with a micro-Raman setup (HR LabRam invers, Jobin–Yvon-Horiba) described in details by Rösch et al. (2005). The spectrometer has an entrance slit of 100 μm and is equipped with a 300 lines mm^−1^ grating. For excitation, 532 nm radiation from a frequency-doubled Nd:YAG laser (Coherent Compass) with a laser power of 10 mW incident on the sample was used. The Raman scattered light was detected by a charge-coupled device (CCD) camera operating at 220 K. A Leica PLFluoar x50/0.5 objective focused the laser light on the samples. For spatially resolved measurements, a *x*–*y* motorized stage (Merzhäuser) with a minimum possible step size of 0.1 μm was used. The maps were recorded with spatial resolution of 0.5 µm in both, *x* and *y*, directions. The *z* direction was fixed during the map recording. For wavenumber calibration 4-acetamidophenol (4AAP) was used as a reference for subsequent data pre-processing. The Raman spectra were baseline corrected using the program LabSpec 5. Five maps were acquired for each fruit stage.

The Raman chemical images were analyzed by both single Raman band imaging and cluster analysis in MATLAB R14 (The MathWorks Inc., Natick, MA, USA). Single Raman band imaging allows the generation of two-dimensional images based on the integral of different Raman bands that are characteristic for different sample components. These single Raman band images were used for a preliminary analysis and for an initial identification and localization of the biopolymers present in the sample. The K-means cluster analysis was used to obtain locations of spatial clusters of chemical components over the sample. K-means algorithm was performed with a squared Euclidean distance metric, in 20 repetitions, each with a new set of initial cluster centroid positions. The algorithm was initialized with seed points randomly selected from the full data set. K-means returned the solution with the lowest value for sums of point-to-centroid distances.

Furthermore, to compare the fruit Raman data with reference spectra the following commercially available compounds were utilized: high methylated (degree of methylation 85 %) and low methylated (degree of methylation 20 %) pectins (Herbstreit and Fox, Neuenbürg, Germany), microcrystalline cellulose (powder, ca. ~20 μm, Sigma Aldrich) and xyloglucan (tamarind, purity >95 %, Megazyme, Bray, Ireland) as the one of the most common hemicelluloses occurring in fruits and vegetables. All these reference chemicals were used without further purification. All Raman spectra were plotted using the OriginPro program (Origin Lab v8.5 Pro, Northampton, USA). The Raman spectra of the pure polysaccharides and the spectra of the cell wall extracted from the chemical maps were normalized to the C–H stretching vibration around 2900 cm^−1^.

### Statistical analysis

Statistica 10.0 (StatSoft, Inc., Tulusa, OK, USA) was used for the descriptive statistical analysis (average values and standard deviations) and for the analysis of the variance (one-way ANOVA) followed by the post hoc Tukey’s honestly significant difference test (HSD) of the chemical analysis results.

## Results

### Cell wall polysaccharides content

**Fig. 1:**
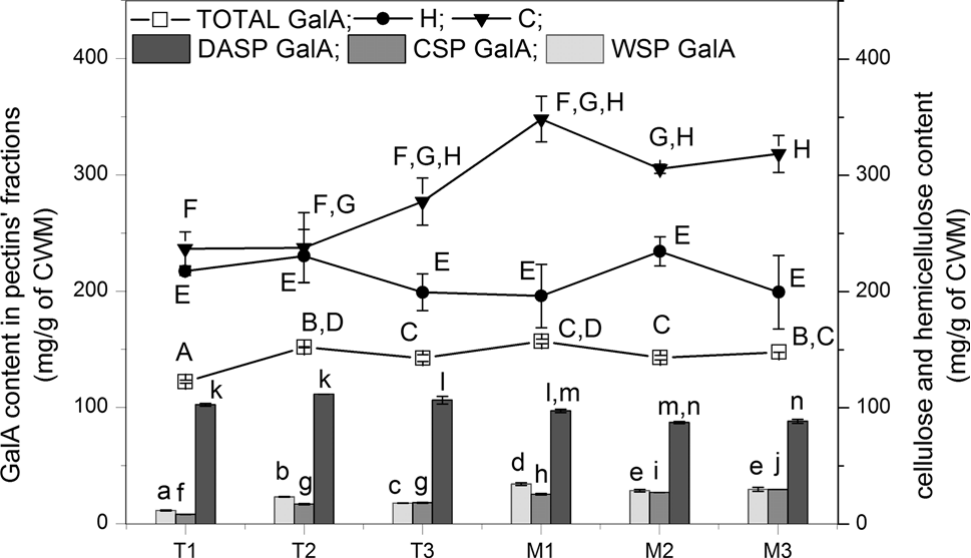
Changes in GalA content in three fractions of pectins (WSP, CSP, DASP) and total value (TOTAL) as well as hemicellulose (H) and cellulose (C) contents are displayed for the apple maturation of Golden Delicious cultivar. The *bars* represent standard deviation. The *same superscript letters* above the bars mean no significant difference at *p* = 0.05. The effect is significant with *p* < 0.05

The cell wall polysaccharide content was evaluated by standard chemical analysis (Fig. 1) (Szymańska-Chargot et al. 2015; Szymanska-Chargot and Zdunek 2013; Cybulska et al. 2015). The GalA content in WSP and CSP fractions increased slowly during the preharvest period, whereas their changes after harvest and during storage were not so significant and the amount of GalA in these fractions was rather stable. Generally, the WSP fraction contained from 11.5 (±0.28) up to 17.82 (±0.09) mg/g of cell wall material dry weight, while the CSP fraction contained from 8.1 (±0.16) to 18.09 (±0.47) mg/g cell wall material dry weight before the harvest date. During the storage the amount of WSP and CSP was rather constant and was around 30 and 17 mg/g of cell wall material dry weight, respectively. In case of the DASP fraction, which was the most abundant in the GalA pectic fraction, an increase of GalA was observed during the fruit maturation (from 102.30 (±1.17) to 106.32 (±3.34) mg/g for T1 and T3, respectively) but during the storage period a slow decrease (from 97.12 (±1.42) to 88.10 (±1.62) mg/g for M1 and M3, respectively) could be observed. Moreover, the content of GalA in DASP was significantly higher as compared to WSP and CSP (Fig. 1).

The changes of the hemicellulose (H) and cellulose (C) content during the investigated time period are also displayed Fig. 1. Generally, the content of hemicellulose in the cell walls oscillated around 200 mg/g of cell wall material dry weight and the time-changes were not statistically significant. At the same time the cellulose content for both cultivars before the harvest was between 236.79 (±14.58) and 277.54 (±20.25) mg/g of cell wall material dry weight. After 1 month storage the cellulose content increased to 348.34 (±19.61) mg/g of cell wall material dry weight and tended to decrease slightly during the next months of storage. Nevertheless, these changes were not statistically significant although the general trend for cellulose was increasing.

### Raman spectra of main plant cell wall polysaccharides

**Fig. 2:**
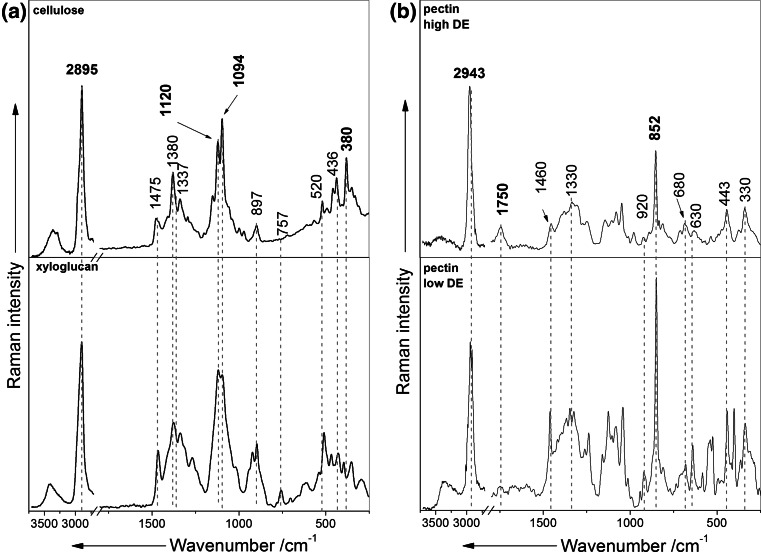
The reference Raman spectra of main plant cell wall polysaccharides: **a** cellulose and xyloglucan, **b** low-esterified (with 20–35 % DE) and high-esterified (with 85 % DE) pectin. *DE* degree of esterification

The reference Raman spectra of the main polysaccharides found in the plant primary cell wall cellulose, xyloglucan (the most abundant hemicellulose in fruits and vegetables) and high and low-esterified pectin are presented in Fig. 2. These Raman spectra are further used for an identification and localization of the main polysaccharides found in cell wall Raman spectra of apples. All the Raman spectra shown in Fig. 2 are characterized by a very strong C–H stretching vibration signal. This C–H stretching band is shifted from 2943 cm^−1^ for high methylated pectin to around 2895 cm^−1^ for cellulose and xyloglucan. Therefore, it is possible to obtain images of the cell wall material (cellulose, pectins, hemicelluloses and lignin, if present) by integrating over this C-H stretching vibration band.

The Raman spectra of cellulose and xyloglucan are very similar due to their similar chemical and structural composition (Fig. 2a). The Raman bands around 1094 and 1120 cm^−1^ are characteristic for C–O–C asymmetric and symmetric stretching vibrations of glycosidic bond in cellulose, respectively (Agarwal 2006; Gierlinger and Schwanninger 2007; Richter et al. 2011). These bands could be found also in the spectrum of xyloglucan where the main backbone consists of β1 → 4-linked glucose residues and is the same as in the cellulose polymer. However, the influence of the hemicelluloses on this band is rather considered to be small (Gierlinger and Schwanninger 2007). Therefore, integrating over these bands could give insight into presence mainly of cellulose with small influence of xyloglucan in the cell wall. The main difference is relative intensity between the bands of both polysaccharides (Himmelsbach et al. 1998).The most characteristic bands for xyloglucan are the bands centered around 757 and 520 cm^−1^ (Fig. 2a). The Raman band around 380 cm^−1^ can be assigned to the β-D-glucosides and is most characteristic for cellulose (Agarwal 2006; Agarwal and Ralph 1997; Chu et al. 2010). But, intensity of this band is very weak the same disqualifying it as the cellulose marker band.

In the cell wall pectins with a different degree of methylesterification can be found. Figure 2b shows the reference Raman spectra of both high and low methylated pectins. The most prominent Raman marker band for the identification of pectin polysaccharides is centered at 852 cm^−1^ which is due to the vibrations of α-glycosidic bonds in pectin. The wavenumber position of this band is shifted from 858 cm^−1^ for a low methylesterification degree to around 842 cm^−1^ for a high methylation degree (Synytsya et al. 2003). This slight wavenumber shift is also visible in the Raman spectra shown in Fig. 2b, although due to an insufficient spectral resolution of the Raman setup (around 9–10 cm^−1^) a clear distinction is rather difficult (Fig. 2b). Nonetheless this band characteristic for pectin shows no overlap with the other plant cell wall polymers and can therefore be used as a marker band (Gierlinger et al. 2013). Another characteristic band for pectins is the C=O stretching vibration of the ester carbonyl group (around 1750 cm^−1^). The presence of this C=O stretch vibration helps to distinguish between pectins with different esterification degree.

### Raman spectra from apple cell wall

**Fig. 3:**
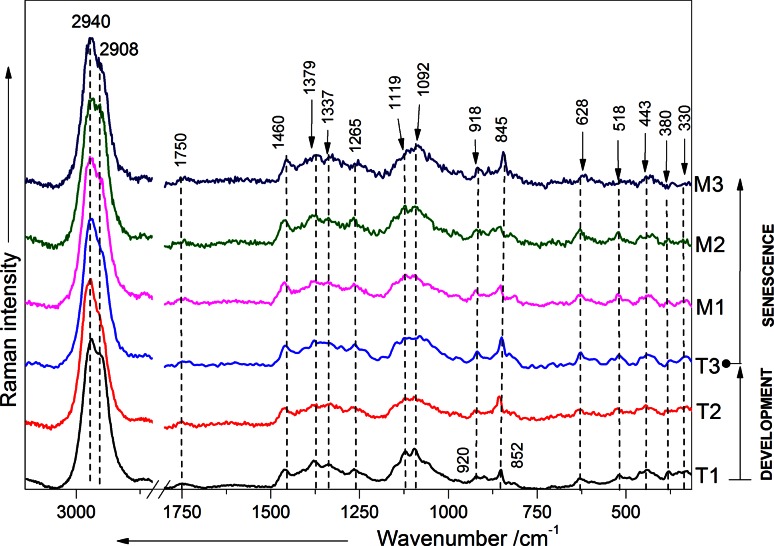
Representative Raman spectra for cell walls of apples in different development stages (*T1*, *T2* and *T3*) and senescence during 1 (*M1*), 3 (*M2*) and 3 (*M3*) months storage

Figure 3 highlights representative Raman spectra recorded in the apple parenchymatic tissue during development and senescence. The Raman spectra of the cell wall in the stages T1, T2 and T3 on first sight seem to be very similar. However, some subtle differences between the spectra are visible, for example, the intensity of some bands has decreased. The most dominant bands are characteristic for mainly cellulose (380, 1092, 1119, 1337, and 1379 cm^−1^) and pectins (330, 443, 852, 918, and 1750 cm^−1^). The only hint for the presence of hemicelluloses is the weak Raman band around 518 cm^−1^ which can be also found in the xyloglucan Raman spectrum (Fig. 2a). It can be clearly seen that the bands centered around 1119 and 852 cm^−1^ have comparable intensities in the case of T2 and T3, whereas for T1 pectin marker band is much lower. The Raman spectrum of the apple cell wall after 1 month storage (M1) did not much change as compared to the spectra recorded for the stages from T1 to T3. However, the most pronounced changes can be found after 2 (M2) and 3 (M3) months of storage. Here, the Raman bands are less resolved, and the relative intensity of the pectin band at ~851 cm^−1^ as compared to the band at 1120–1092 cm^−1^ and characteristic mainly for cellulose is much lower. The band characteristic for the vibrations of α-glycosidic bonds in pectin is shifted to lower wavenumbers when comparing T1 (851 cm^−1^) and M3 (845 cm^−1^). The ester carbonyl group vibration at 1750 cm^−1^ seemed to be stable from T1 to M3. Whereas a band characteristic cellulose centered around 380 cm^−1^ has the tendency to decrease during development and senescence.

### Raman images of cell wall polysaccharides

**Fig. 4:**
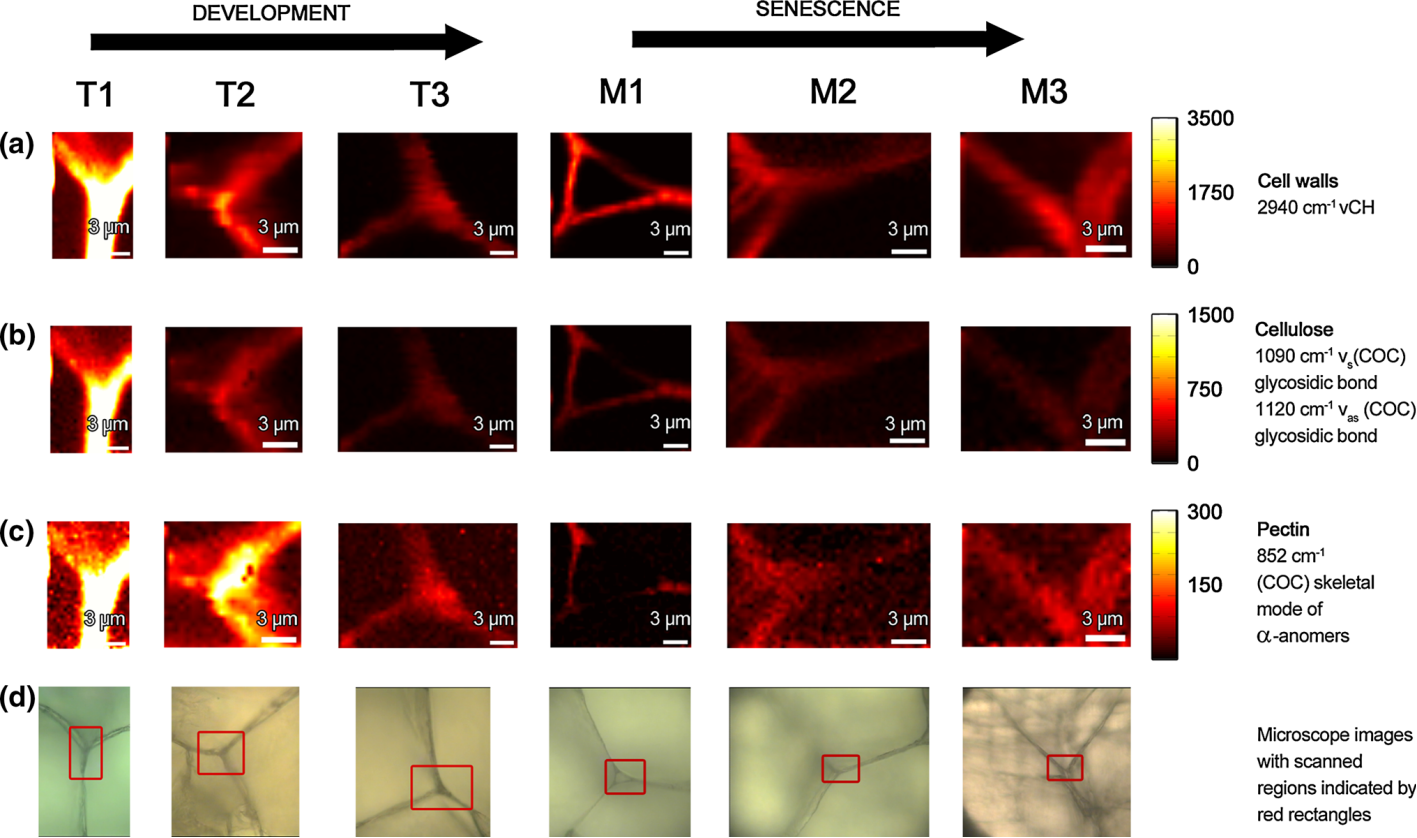
Raman maps of the cell wall in apple parenchyma tissue at the development stages *T1*, *T2* and *T3* and during 1 *M1*, 2 *M2* and 3 *M3* months storage. The Raman maps were obtained by integrating Raman bands from 2760 to 3100 cm^−1^ (C–H stretching vibration) (cell wall material CW) (**a**), from 1000 to 1179 cm^−1^ (mainly cellulose) (**b**), from 840 to 885 cm^−1^ (pectin) (**c**). The *red squares* on the bright field images (**d**) mark the investigated areas by Raman imaging

The chemical Raman maps calculated by integrating over selected wavenumber ranges corresponding to the Raman bands characteristic for individual cell wall polysaccharides are presented in Fig. 4. To remove the interfering background all sample point integrals were restricted using fixed, arbitrary chosen values to obtain binary masks. After this preprocessing only sample points from the cell wall remained. Here, we could monitor changes in the apple cell wall composition during the development process as well as the 3 months storage with a simultaneous spatial localization of these changes. In case of apples the most pronounced changes are connected with pectins that undergo an enzymatic digestion. Therefore, the Raman maps were mainly generated for the area of the cell wall corners rich in pectin. The chemical images of the cell wall components (i.e., cellulose, hemicellulose and pectin) (Fig. 4a, b) were obtained by integrating over the band with a maximum at 2940 cm^−1^, reflecting the CH-stretching vibrations, which are characteristic for all primary cell wall polysaccharides (Richter et al. 2011). The cellulose distribution was obtained by integration over an area represented by the two Raman bands at 1090 and 1120 cm^−1^ (Fig. 4a, b), which can be assigned to the stretching vibration of the glycosidic bond in cellulose. These bands are characteristic for cellulose oriented in parallel and perpendicular to the excitation light, respectively (Gierlinger et al. 2008). The integration over these wavenumber regions displays the cellulose which is evenly distributed over the investigated cell walls (Fig. 4b). However, it must be remembered that these bands are influenced also by the bands originating from hemicelluloses. The peak at 851 cm^−1^ (Fig. 3) corresponds to the band for the (COC) skeletal mode of α-anomers in pectin (Gierlinger et al. 2008). An integration over this band confirmed higher concentrations of pectin within the cell wall, but the highest intensity was found close to the cell corner of the apple parenchyma (Fig. 4c). The cell corners and middle lamella are particularly enriched in pectin, especially homogalacturonan. The compounds of this part of the tissue are responsible for cell–cell adhesion (Marry et al. 2006). Due to the fact that the sample’s components are homogeneously localized in the studied cell wall, high concentrations of both pectin and cellulose were found in similar locations. Unfortunately, it was impossible to localize the hemicellulose in cell walls of apple tissue. The chemical structure of hemicellulose is very similar to cellulose (Fig. 2a) and the band characteristic for hemicellulose overlapped with the band characteristic for cellulose. Moreover, it seems that this group of compounds, i.e., hemicelluloses was distributed homogeneously in the cell wall. The other reason why the spatial distribution of hemicelluloses could not be visualized is their small amount in the cell wall material. Similar conclusions were drawn previously for tomato pericarp tissue (Chylinska et al. 2014).

During development of the apple its cell wall contains large amounts of pectins which are uniformly distributed along with other non-pectic polysaccharides—cellulose and hemicellulose (Fig. 4b T1, T2, T3). However, the localization of pectins is changing from homogenously distributed (Fig. 4c T1) to more concentrated in the cell wall corners for the mature apple fruit (Fig. 4c T3).

Figure 4 M1, M2 and M3 presents Raman images of the apple cell walls during 3 months storage. Still the main components of the cell walls are non-pectic polysaccharides: cellulose and hemicellulose (Fig. 4b M1, M2 and M3). In the case of sample M1 pectic polysaccharides can be found in the whole cell wall with significantly higher amounts in cell wall corners. After 2 months (M2) and 3 months (M3) the storage pectic polysaccharides seem to be dispersed evenly with the difference that after 3 months a substantial decrease could be observed.

**Fig. 5:**
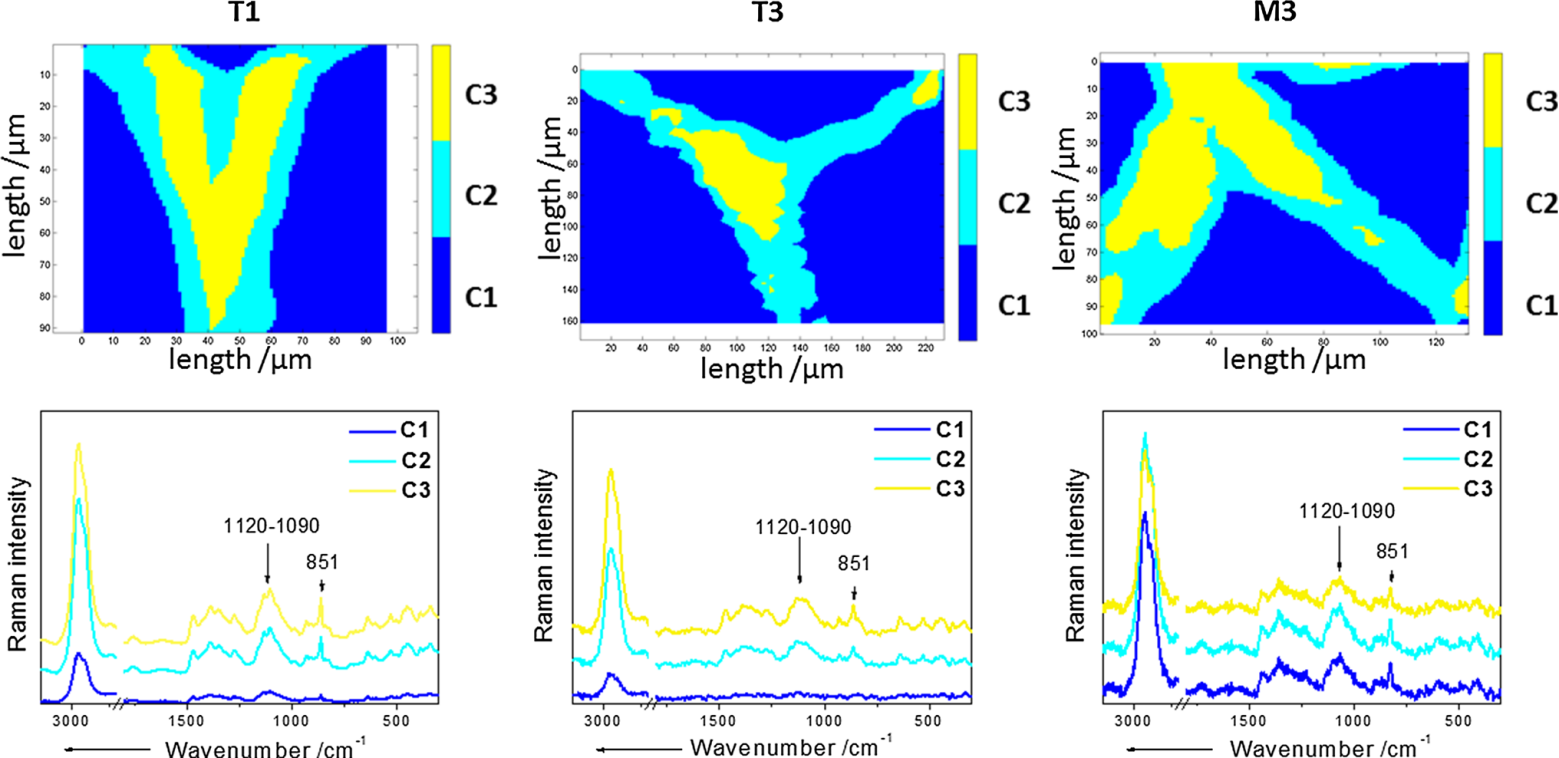
K-means clustering results for Raman maps of an apple parenchyma tissue cell wall–cluster map (*upper row*) and corresponding cluster average spectra for these methods (*bottom row*). The most characteristic Raman bands are highlighted (for their assignments it is referred to the text). For simplicity only the stages *T1*, *T3* and *M3* are shown: C1 (*yellow cluster*)—middle lamella, mostly pectins; C2 (*blue cluster*)—cell wall, mostly cellulose and hemicellulose; C3 (*dark blue*)—background

The cluster analysis of the Raman maps is shown in Fig. 5. The aim of the cluster analysis is to group analyzed objects (spectra from the map) into clusters, so that objects (spectra) most similar to each other belong to the same cluster. The K-means cluster maps provide detailed information about changes in the distribution of cell wall polysaccharides. Cluster C1 is located in the middle part of adjacent cell walls, whereas cluster C2 is located at the edges of the cell wall. Cluster C3 is due to the background located in the cell lumen which is supported by the mean spectrum of this cluster for all samples. The mean spectrum of cluster C1 has a sharp and intensive band centered at 852 cm^−1^, characteristic for pectic polysaccharide. However, also, bands characteristic for cellulose (1120–1090 cm^−1^) are present. The mean spectrum of cluster C2 is similar to the mean spectrum of C1, but the relative intensity of the cellulose band is higher than that of the pectin band, which implicates that the C2 cluster is probably composed of all cell wall polysaccharides. This leads to the conclusion that the yellow cluster C1 can be mainly connected with the middle lamella region and the light blue cluster C2 with the cell wall. From Fig. 5 it can be concluded that from T1 to T3 the area occupied by the cluster C1 is decreasing to concentrate in the tricellular junction. For the sample M3 the cluster C1 again increases in its area. In addition, the differences between the mean spectra of the clusters C1 and C2 are minimal (Fig. 5 M3).

## Discussion

Until now changes in the distribution of cell wall polysaccharides have been highlighted using the immunolabelling method (Ng et al. 2013, 2015). However, this method is relatively expensive and the complicated sample preparation procedure is required, but on the other hand is very selective and has this great advantage that localization of each polysaccharide is possible. Here, we report about recording the polysaccharide distribution in apple parenchyma cell walls using confocal Raman microscopy. This method is non-destructive and does not require extensive sample pretreatment. Moreover, an advantage of Raman microspectroscopy is the possibility of acquiring the complete information about the spatial distribution of chemical components during a single measurement in form of a hyperspectral image (Schmidt et al. 2010; Gierlinger et al. 2012).

Raman images of apple cell walls were generated by integrating over a certain wavenumber region highlighting substantial changes in the amount and localization of the main cell wall polysaccharides. Due to the sharp Raman band around the 852 cm^−1^ changes in the pectin content and distribution can be visualized, whereas integrating over the Raman bands around 1090–1120 cm^−1^ depicts the distribution of cellulose. It was reported that by integrating over Raman bands around 1736 cm^−1^ or in the range of 874–934 cm^−1^ highlights the hemicellulose distribution, however, in this study these bands were overlapping with other bands making the imaging of the hemicellulose distribution in apple cell walls impossible (Gierlinger et al. 2008).

During the apple development and senescence the most important changes take place in middle lamella and tricellular junctions which are particularly abound in the pectic polysaccharide. It could be shown that the pectin distribution changed from dispersed along the cell wall and mixed evenly with cellulose/hemicellulose (T1—6 weeks before harvest) to concentrated in cell wall corners and middle lamella at the harvest stage (T3). Whereas during postharvest senescence the pectin amount decreased and after 2-month cold storage was again evenly dispersed along the cell wall. Ng et al. (2013) proved using immunolabelling with the monoclonal antibodies specific for the non-esterified (LM 19) and esterified (LM 20) homogalacturonan that during the ripening process of apples the pectins are decreasing in the intercellular junctions, but are still present in the middle lamella region which lead to an increase of the air spaces (Ng et al. 2013). The decrease in the overall pectic content was reported along with the apple fruit senescence during postharvest storage (Billy et al. 2008; Gwanpua et al. 2014).

The pectic content is usually evaluated using chemical methods. Pectins are extracted from plant material using different media (water, calcium chelator or diluted alkali) with respect to their chemical bonding in the cell wall. WSP are rather weakly bonded to the other cell wall components. Whereas, chelator extracts the Ca-bridges from pectins which are held together via ionic interactions and lead to contributions to the CSP fraction. The CSP fraction is especially highly abundant in the middle lamella (Matar and Catesson 1988). The last fraction of pectins which are soluble in sodium carbonate (DASP) are bonded via inter polymeric ester bonds in the cell wall. During maturation and senescence the pectins undergo depolymerisation and deestryfication. It was also reported that the divalent cationic interaction undergoes degradation (the decrease in GalA in CSP fraction) which leads to the middle lamella dissolution (Prasanna et al. 2007).

Chemical analyses showed that the hemicellulose content was constant in time whereas cellulose was increasing until harvest time. Chemical analysis in the experiments reported here showed also that the total amount of GalA is constant during the pre- and postharvest period (Billy et al. 2008). While GalA extracts in fractions of covalently bounded pectins (DASP) decreases, the GalA content increases for the fractions containing an ionic bounded CSP and loosely bonded WSP. Similar results were shown previously by both chemical (Bartley and Knee 1982; Gwanpua et al. 2014; Zdunek et al. 2015) and atomic force microscopy (Cybulska et al. 2015; Paniagua et al. 2014) experiments. Our experiment showed that Raman microspectroscopy provides new insight into the time and spatial changes of pectins that, due to the best of our knowledge, has not been shown before. Raman images revealed that the most pronounced changes connected with pectins occurred in the cell corners zones suggesting that fruits at harvest time secrete pectins in the junction corners to ensure mechanical resistance of tissue while both in the preharvest and postharvest period the pectin distribution is rather homogeneous. However, it must be noted that due to limited spatial resolution of Raman imaging it is still not possible to distinguish between the middle lamella zone and the primary cell wall zone thus comprehensive interpretation is still limited.

## Conclusions

The course of changes in the cell wall composition of apple parenchymatic tissue during on-tree maturation and postharvest senescence was followed using Raman imaging. The obtained results showed that Raman spectroscopy and especially Raman imaging is a very useful technique for the identification of compositional changes in plant tissue during their development. In the case of apples tissue the main changes were connected with pectic polysaccharides. During on-tree development, the pectin distribution changed from polydispersed in cell wall to cumulated in cell wall corners. During apple storage, after 3 months, the pectin distribution returned to evenly dispersed along the cell wall. These findings represent an important benefit as compared to standard chemical analysis that does not allow spatial evaluation. Comparing to immunolabelling methods, that were not used here, Raman imaging benefits in terms of cost and time effectiveness.

### *Author contribution statement*

MS-C designed experiment, performed Raman experiment, interpreted data, and wrote the manuscript; MC prepared CWM samples, analyzed polysaccharide content; PMP prepared the image analysis software for Raman maps; PR took part in Raman maps acquisition, data interpretation and manuscript preparation; MS, JP & AZ helped in data interpretation and manuscript preparation. All authors read and approved the manuscript.

## Electronic supplementary material

Below is the link to the electronic supplementary material.

Fig. S1. Raman maps of the cell wall in apple parenchyma tissue at the development stages T1, T2 and T3 and during one M1, two M2 and three M3 months storage. The Raman maps were obtained by integrating Raman bands from 1000 cm^−1^ to 1179 cm^−1^ (mainly cellulose) (a) and from 840 cm^−1^ to 885 cm^−1^ (pectin) (b) (TIFF 796 kb)

## References

[CR1] Agarwal UP (2006). Raman imaging to investigate ultrastructure and composition of plant cell walls: distribution of lignin and cellulose in black spruce wood (*Picea mariana*). Planta.

[CR2] Agarwal UP, Ralph SA (1997). FT-Raman spectroscopy of wood: identifying contributions of lignin and carbohydrate polymers in the spectrum of black spruce. Appl Spectro.

[CR3] Bartley IM, Knee M (1982). The chemistry of textural changes in fruit during storage. Food Chem.

[CR4] Billy L, Mehinagic E, Renard CMGC, Prost C (2008). Relationship between texture and pectin composition of two apple cultivars during storage. Postharvest Biol Technol.

[CR5] Carpita NC, Gibeaut DM (1993). Structural models of primary cell walls in flowering plants: consistency of molecular structure with the physical properties of the walls during growth. Plant J.

[CR6] Chu L-Q, Masyuko R, Sweedler JV, Bohn PW (2010). Base-induced delignification of *Miscanthus × Giganteus* studied by three-dimensional confocal Raman imaging. Bioresour Tech.

[CR7] Chylińska M, Szymańska-Chargot M, Zdunek A (2014). Imaging of polysaccharides in the tomato cell wall with Raman microspectroscopy. Plant Methods.

[CR8] Chylińska M, Szymańska-Chargot M, Kruk B, Zdunek A (2016). Study on dietary fiber by Fourier transform-infrared spectroscopy and chemometric methods. Food Chem.

[CR9] Cosgrove DJ, Jarvis MC (2012). Comparative structure and biomechanics of plant primary and secondary cell walls. Front Plant Sci.

[CR10] Cybulska J, Zdunek A, Kozioł A (2015). The self-assembled network and physiological degradation of pectins in carrot cell walls. Food Hydrocoll.

[CR11] Gierlinger N, Schwanninger M (2007). The potential of Raman microscopy and Raman imaging in plant research. Spectrosc Int J.

[CR12] Gierlinger N, Sapei L, Paris O (2008). Insights into the chemical composition of Equisetum hyemale by high resolution Raman imaging. Planta.

[CR13] Gierlinger N, Keplinger T, Harrington M (2012). Imaging of plan cell walls by confocal Raman microscopy. Nat Protoc.

[CR14] Gierlinger N, Keplinger T, Harrington M, Schwanninger M (2013) Raman imaging of lignocellulosic feedstock. Chapter 8 in “Cellulose–Biomass Conversion” edited by Theo van de Ven and John Kadla, ISBN 978-953-51-1172-6, InTech

[CR15] Gwanpua SG, Van Buggenhout S, Verlinden BE, Christiaens S, Shpigelman A, Vicent V, Kermani ZJ, Nicolai BM, Hendrickx M, Geeraerd A (2014). Pectin modifications and the role of pectin-degrading enzymes during postharvest softening of Jonagold apples. Food Chem.

[CR16] Hamann T (2012). Plant cell wall integrity maintenance as an essential component of biotic stress response mechanisms. Front Plant Sci.

[CR17] Harker FR, Redgwell RJ, Hallett IC, Murray SH, Carter G (2010) Texture of fresh fruit, in horticultural reviews, volume 20. In: Janick J (ed), John Wiley & Sons, Inc., Oxford, UK

[CR18] Himmelsbach DS, Khahili S, Akin DE (1998). Near-infrared Fourier-transform Raman spectroscopy of flax (*Linum usitatissimum* L.) stems. J Agric Food Chem.

[CR19] Jarvis MC, Briggs SPH, Knox JP (2003). Intercellular adhesion and cell separation in plants. Plant Cell Environ.

[CR20] Konopacka D, Jesionkowska K, Kruczyńska D, Stehr R, Schoorl F, Buehler A, Egger S, Codarin S, Hilaire C, Höller I, Guerra W, Liverani A, Donati F, Sansavini S, Martinelli A, Petiot C, Carbó J, Echeverria G, Iglesias I, Bonany J (2010). Apple and peach consumption habits across European countries. Appetite.

[CR21] Li Z, Yang H, Li P, Liu J, Wang J, Xu Y (2013). Fruit biomechanics based on anatomy: a review. Int Agrophys.

[CR22] Marry M, Roberts K, Jopson SJ, Huxham IM, Jarvis MC, Corsar J, Robertson E, McCann MC (2006). Cell–cell adhesion in fresh sugar beet root parenchyma requires both pectin esters and calcium cross-links. Phys Plant.

[CR23] Matar D, Catesson AM (1988). Cell plate development and delayed formation of the pectic middle lamella in root meristems. Protoplasma.

[CR24] Ng JK, Schröder R, Sutherland PW, Hallett IC, Hall MI, Prakash R, Smith BG, Melton LD, Johnston JW (2013). Cell wall structures leading to cultivar differences in softening rates develop early during apple (*Malus* *×* *domestica*) fruit growth. BMC Plant Biol.

[CR25] Ng JK, Schröder R, Brummell DA, Sutherland PW, Hallett IC, Smith BG, Melton LD, Johnston JW (2015). Lower cell wall pectin solubilisation and galactose loss during early fruit development in apple (Malus *×* domestica) cultivar ‘Scifresh’ are associated with slower softening rate. J Plant Physiol.

[CR26] Paniagua C, Pose S, Morris VJ, Kirby AR, Quesada MA, Mercado JA (2014). Fruit softening and pectin disassembly: an overview of nanostructural pectin modifications assessed by atomic force microscopy. Ann Bot.

[CR27] Prasanna V, Prabha TN, Tharanathan RN (2007). Fruit ripening phenomena—an overview. Crit Rev Food Sci.

[CR28] Qin J, Chao K, Kim MS (2011). Investigation of Raman chemical imaging for detection of lycopen changes in tomatoes during postharvest ripening. J Food Eng.

[CR29] Redgwell RJ, Curti D, Gehin-Delval C (2008). Physicochemical properties of cell wall materials from apple, kiwifruit and tomato. Eur Food Res Technol.

[CR30] Renard CMGC (2005). Variability in cell wall preparations: quantification and comparison of common methods. Carbohydr Polim.

[CR31] Richter S, Mussig J, Gierlinger N (2011). Functional plant cell wall design revealed by the Raman imaging approach. Planta.

[CR32] Rösch P, Harz M, Schmitt M, Popp J (2005). Raman spectroscopic identification of single yeast cells. J Raman Spectrosc.

[CR33] Schmidt M, Schwartzberg AM, Perrera PN, Weber-Bargioni A, Carroll A, Sarkar A, Bosneaga E, Urban JJ, Song J, Balakshin MY, Capanema EA, Auer M, Adams PD, Chiang VL, Schuck PJ (2009). Label-free in situ imaging of lignification in the cell wall of low transgenic *Populus trichocarpa*. Planta.

[CR34] Schmidt M, Schwartzberg AM, Carroll A, Chaibang A, Adams PD, Schuck PJ (2010). Raman imaging of cell wall polymers in *Arabidopsis thaliana*. Biochem Biophys Res Commun.

[CR35] Schulz H (2008) Modern techniques for food authentication. In: Sun Da-Wen (ed) Spectroscopic technique: Raman spectroscopy, Chapter 5, Academic Press, Elsevier

[CR36] Strehle MA, Rösch P, Baranska M, Schulz H, Popp J (2005). On the way to a quality control of the essential oil of fennel by means of Raman spectroscopy. Biopolymers.

[CR37] Synytsya A, Copikova J, Matejka P, Machovic V (2003). Fourier transform Raman and infrared spectroscopy of pectins. Carbohydr Polym.

[CR38] Szymańska-Chargot M, Zdunek A (2013). Use of FT-IR spectra and PCA to the bulk characterization of cell wall residues of fruits and vegetables along a fraction process. Food Biophys.

[CR39] Szymańska-Chargot M, Chylińska M, Kruk B, Zdunek A (2015). Combining FT-IR spectroscopy and multivariate analysis for qualitative and quantitative analysis of the cell wall composition changes during apples development. Carbohydr Polim.

[CR40] Volz RK, Harker FR, Lang S (2003). Firmness decline in gala apple during fruit development. J Am Soc Hortic Sci.

[CR41] Winisdorffer G, Musse M, Quellec S, Barbacci A, Le Gall S, Mariette F, Lahaye M (2015). Analysis of the dynamic mechanical properties of apple tissue and relationships with the intracellular water status, gas distribution, histological properties and chemical composition. Postharvest Biol Technol.

[CR42] Xia Y, Petti C, Williams MA, DeBolt S (2014). Experimental approaches to study plant cell walls during plant-microbe interactions. Front Plant Sci.

[CR43] Zdunek A, Kozioł A, Pieczywek PM, Cybulska J (2014). Evaluation of the nanostructure of pectin, hemicellulose and cellulose in the cell walls of pears of different texture and firmness. Food Bioprocess Tech.

[CR44] Zdunek A, Kozioł A, Cybulska J, Lekka M, Pieczywek PM (2015). The stiffening of the cell walls observed during physiological softening of pears. Planta.

