# Supplementary figures and images for: Raman imaging of changes in the polysaccharides distribution in the cell wall during apple fruit development and senescence
Source: Planta. 2016 Jan 5;243:935–45. doi: 10.1007/s00425-015-2456-4 (PMC4819746; doi:10.1007/s00425-015-2456-4)

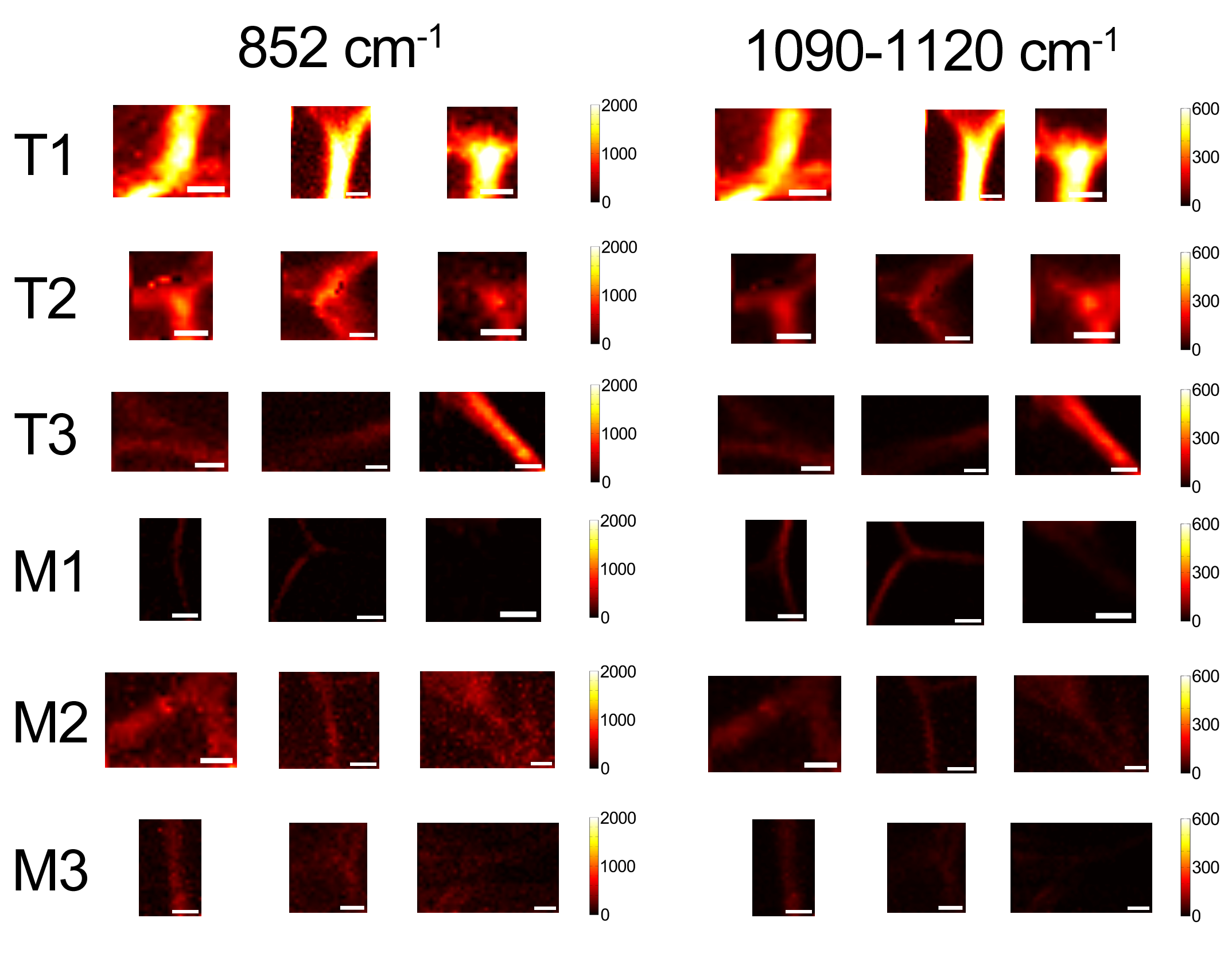

Supplement: Supplementary file 1 — Fig. S1. Raman maps of the cell wall in apple parenchyma tissue at the development stages T1, T2 and T3 and during one M1, two M2 and three M3 months storage. The Raman maps were obtained by integrating Raman bands from 1000 cm−1 to 1179 cm−1 (mainly cellulose) (a) and from 840 cm−1 to 885 cm−1 (pectin) (b) (TIFF 796 kb) [file 425_2015_2456_MOESM1_ESM.tif]
